# Möbius Transforms, Cycles and *q*-triplets in Statistical Mechanics

**DOI:** 10.3390/e21121155

**Published:** 2019-11-26

**Authors:** Jean Pierre Gazeau, Constantino Tsallis

**Affiliations:** 1 Astroparticles and Cosmology (UMR 7164), Sorbonne Paris Cité, Univ Paris Diderot, 75205 Paris, France; gazeau@apc.in2p3.fr; 2 Centro Brasileiro de Pesquisas Físicas and National Institute of Science and Technology of Complex Systems, Rua Xavier Sigaud 150, Rio de Janeiro 22290-180, Brazil; 3 Santa Fe Institute, 1399 Hyde Park Road, Santa Fe, NM 87501, USA; 4 Complexity Science Hub Vienna, Josefstädter Strasse 39, 1080 Vienna, Austria

**Keywords:** non-additive entropy, q-statistics, Möbius transform, complex systems

## Abstract

In the realm of Boltzmann-Gibbs (BG) statistical mechanics and its *q*-generalisation for complex systems, we analysed sequences of *q*-triplets, or *q*-doublets if one of them was the unity, in terms of cycles of successive Möbius transforms of the line preserving unity (q=1 corresponds to the BG theory). Such transforms have the form q↦(aq+1−a)/[(1+a)q−a], where *a* is a real number; the particular cases a=−1 and a=0 yield, respectively, q↦(2−q) and q↦1/q, currently known as additive and multiplicative dualities. This approach seemingly enables the organisation of various complex phenomena into different classes, named *N*-complete or incomplete. The classification that we propose here hopefully constitutes a useful guideline in the search, for non-BG systems whenever well described through *q*-indices, of new possibly observable physical properties.

## 1. Introduction

Together with Maxwell electromagnetism, and classical, quantum and relativistic mechanics, Boltzmann–Gibbs (BG) statistical mechanics constitutes a pillar of contemporary theoretical physics. This powerful theory is based on the optimisation, under simple appropriate constraints, of the (additive) BG entropic functional $S_{BG}$, where $S_{BG}=k\sum_{i=1}^{W}p_{i}\ln\left(1/p_{i}\right)$ (with $\sum_{i=1}^{W}p_{i}=1$, *k* being a conventional positive constant chosen once and forever) for a simple discrete system with *W* microscopic possibilities whose occurrence probabilities are given by $\left\{p_{i}\right\}$. This fact typically leads, for a nonlinear (conservative or dissipative), dynamic, classical system with positive maximal Lyapunov exponent, to a (asymptotically) linear time growth of $S_{BG}\left(\left\{p_{i}\left(t\right)\right\}\right)$ while occupying a finely partitioned phase space. It also leads, for nearly all initial conditions for quite generic BG systems, to an exponential time relaxation towards its stationary state. Finally, this stationary state (usually referred to as *thermal equilibrium*) is characterised by the celebrated BG exponential weight $p_{i}=e^{-E_{i}/kT}/Z_{BG}$ with its partition function given by $Z_{BG}\equiv \sum_{j=1}^{W}e^{-E_{j}/kT}$, $E_{j}$ being the energy eigenvalue of the *j*-th state of a quantum conservative Hamiltonian system with specific boundary conditions.

For vast classes of complex natural, artificial and social systems, this relatively simple scenario fails. More precisely, either it is discrepant from experimental, observational or computational evidences, or it is plainly not calculable (i.e., mathematically ill-defined), typically because its partition function $Z_{BG}$ diverges, as already alerted long ago by Gibbs himself [1]. Consequently, a generalisation of the BG theory becomes mandatory. Such a generalisation was proposed in 1988 [2,3,4], and has been useful for wide classes of complex systems; e.g., cold atoms [5,6], high-energy collisions of elementary particles [7,8], granular matter [9], low-dimensional maps [10], asymptotically scale-free networks [11] and cosmic rays [12], to quote but a few of them. This generalisation consists of optimising, under appropriate simple constraints, a nonadditive entropy $S_{q}$ through the introduction of a deformation parameter *q*; namely,

(1) $$S_{q}=k\sum_{i=1}^{W}p_{i}\ln_{q}\frac{1}{p_{i}}=-k\sum_{i=1}^{W}p_{i}\ln_{2-q}\frac{1}{p_{i}}\;\;\;\left(q\in R\;;\;S_{1}=S_{BG}=-k\sum_{i=1}^{W}p_{i}\ln p_{i}\right)\;,$$

with $\ln_{q}z\equiv \frac{z^{1-q}-1}{1-q}\;\;\left(\ln_{1}z=\log z\;;\;z\in C\right)$; its inverse function is given by $e_{q}^{z}={\left[1+\left(1-q\right)z\right]}^{1/(1-q)}$. The fact that $S_{q}$ is generically nonadditive is straightforwardly verified, more precisely as follows:

(2) $$\frac{S_{q}\left(A+B\right)}{k}=\frac{S_{q}\left(A\right)}{k}+\frac{S_{q}\left(B\right)}{k}+\left(1-q\right)\frac{S_{q}\left(A\right)}{k}\frac{S_{q}\left(B\right)}{k},$$

where *A* and *B* are *probabilistically independent*; i.e., $p_{ij}^{A+B}=p_{i}^{A}p_{j}^{B},\;\forall \left(i,j\right)$. This property recovers, for q=1, the well known additivity of the $S_{BG}$ functional.

For wide classes of nonlinear dynamical systems with *zero*, instead of a positive, maximal Lyapunov exponent, it turns out that the linear time growth occurs for $S_{q_{entropy}}$ with $q_{entropy}<1$. Concomitantly, relaxation occurs $q_{relaxation}$-exponentially with $q_{relaxation}>1$ towards a stationary state characterised by $p_{i}=e_{q_{energy}}^{-E_{i}/kT}/Z_{q_{energy}}$ with the partition function given by $Z_{q_{energy}}\equiv \sum_{j=1}^{W}e_{q_{energy}}^{-E_{j}/kT}$ with $q_{energy}\neq 1$ [2,3,4].

The situation is sometimes more complex than just described. For instance, the distribution of momenta of a many-body Hamiltonian system usually follows a $q_{moment}$-Gaussian form with $q_{moment}\neq 1$ not necessarily coincident with $q_{energy}$. The general scenario is, for a given complex system, that we may have an infinite countable set of different *q*’s, corresponding to different one-body or many-body properties. However, only a small number of these *q*’s are in principle independent, all the others being related to those few through relatively simple analytic relations. The whole scenario appears to be strongly reminiscent of the scaling relations existing between the various exponents that emerge in the theory of critical phenomena (e.g., α+2β+γ=2, (2−η)ν=γ, dν=2−α and similar ones).

The present work constitutes an attempt to formally establish, at least for some important classes of systems, the relations between the various *q*’s that are necessary to fully characterise the universality classes associated with a given complex system. In this attempt we follow along the lines of [13,14,15,16,17,18,19,20,21]. The needed algebraic and geometric material is described in Section 2 in terms of SU(1,1)∼ SL(2,R) homographic group actions on the unit disk (respectively upper half-plane) [22,23]. In Section 3 we restrict these actions to particular ones, leaving some point fixed, and study the corresponding subsets of the groups. In Section 4 we proceed with the analysis of two-term or three-term cycles in view of displaying some universal relationship when they involve a doublet or a triplet of parameters *q*. The same is carried out in Section 5 in terms of alternative variables. Numerical examples extracted from various observations are analysed in Section 6 from the point of view of such universality rules. Final discussion and comments constitute the content of Section 7.

## 2. Unit Disk, Circle, Half-Plane and Real Line: A Reminder

Since specific conformal or homographic transformations of the real line occupy the central role in the present work, we think useful to give an overview of its mathematical context. More details can be found in chapters VI and VII of [22] or in Chapter 8 in [23].

The (open) unit disk in the complex plane is defined as

(3) $$D\overset{\mathrm{def}}{=}\left\{z\in C\;,\;|z|<1\right\}\;.$$

Besides the unit disk, there is another equivalent representation commonly used in two-dimensional hyperbolic geometry and defining a model of hyperbolic space on the upper half-plane; namely, the Poincaré half-plane. The disk D and the upper half plane P+={Z∈C,ImZ>0} are related by a conformal map, called Möbius transformation,

(4) $$P_{+}∋Z\mapsto z=e^{i\phi }\;\frac{Z-Z_{0}}{Z-{\bar{Z}}_{0}}\in D\;,$$

ϕ and $Z_{0}$ being arbitrary, and $\bar{Z}$ is the complex conjugate of *Z*. The canonical mapping is given by $Z_{0}=i$ and ϕ=π/2. It takes i to the centre of the disk and the origin *O* to the bottom of the disk.

As the sphere $S^{2}$ is invariant under space rotations forming the group SO(3)≃SU$\left(2\right)/Z_{2}$, $Z_{2}=\left\{1,-1\right\}$, the unit disk D is invariant under transformations of the *homographic* or Möbius type:

(5) $$D∋z\mapsto z^{\prime }=\left(\alpha \;z+\beta \right)\;{\left(\bar{\beta }\;z+\bar{\alpha }\right)}^{-1}\in D\;,$$

with α, β∈C and ${\left|\alpha \right|}^{2}-{\left|\beta \right|}^{2}\neq 0$. Since a common factor of α and β is unimportant in the transformation (5), one can associate to the latter the 2×2 complex matrix

(6) $$\left(\begin{array}{cc} \alpha & \beta \\ \bar{\beta } & \bar{\alpha } \end{array}\right)\overset{\mathrm{def}}{=}g\;,\;\mathrm{with}\;\det g={\left|\alpha \right|}^{2}-{\left|\beta \right|}^{2}=1\;,$$

and we will write $z^{\prime }=g\cdot z$. These matrices form the group SU(1,1), one of the simplest examples of a simple, non-compact Lie group. It should be noticed that SU(1,1) leaves invariant the boundary $S^{1}≃$ U(1) of D under the transformations (5).

Let us turn our attention to the corresponding symmetries in the Poincaré half-plane. The conversion is carried out through a simple multiplication of matrices involving the specific (or “canonical“) Möbius transformation, written here as

(7) $$z=\frac{Z-i}{1-iZ}\equiv \frac{1}{\sqrt{2}}\;\left(\begin{array}{cc} 1 & -i\\ -i & 1 \end{array}\right)\cdot Z\equiv m\;\cdot Z\;,\;Z=m^{-1}\cdot z\;,$$

and conversely

(8) $$Z=\frac{z+i}{iz+1}\;.$$

Note that when extended to the boundaries, the bijection 7 is a Cayley transformation that maps in a stereographic way, the unit circle $S^{1}$ onto the real line

(9) $$S^{1}∋e^{i\theta }\mapsto t=\frac{e^{i\theta }+i}{i\;e^{i\theta }+1}\in R\;,\;\theta \in \left[0,2\pi \right)\;,$$

where θ=0↦t=1, θ=π/2↦t=∞, θ=π↦t=−1 and $\theta =\frac{3\pi }{2}\mapsto 0$. Conversely,

(10) $$R∋t\mapsto e^{i\theta }=\frac{t-i}{-i\;t+1}\in S^{1}\;.$$

Therefore, the transformation $z^{\prime }=g\cdot z$ where g∈ SU(1,1) becomes in the half-plane

(11) $$Z^{\prime }=s\cdot Z=\frac{aZ+b}{cZ+d}\;,$$

with

(12) s=m−1gm=Reα+ImβImα+Reβ−Imα+ReβReα−Imβ≡abcd,a,b,c,d∈R,

and conversely,

(13) $$g=m\;s\;m^{-1}=\frac{1}{2}\left(\begin{array}{cc} a+d+i(b-c) & b+c+i(a-d)\\ b+c-i(a-d) & a+d-i(b-c) \end{array}\right)\equiv \left(\begin{array}{cc} \alpha & \beta \\ \bar{\beta } & \bar{\alpha } \end{array}\right)\;,\;\alpha ,\;\beta \in C\;.$$

Since dets=1, the set of such 2×2 real matrices form the group SL(2,R), which leaves invariant the upper half-plane and its boundary, which is the real line R.

Indeed, the action (5) of $g=\left(\begin{array}{cc} \alpha & \beta \\ \bar{\beta } & \bar{\alpha } \end{array}\right)\in SU\left(1,1\right)$ on D extends to the boundary as

(14) $$g\cdot e^{i\theta }=\left(\alpha \;e^{i\theta }+\beta \right)\;{\left(\bar{\beta }\;e^{i\theta }+\bar{\alpha }\right)}^{-1}\equiv e^{i\theta^{\prime }}\in S^{1}\;,$$

and so leaves the latter invariant. Similarly, SL(2,R) acts homographically on the real line R as

(15) $$R∋q\mapsto s\cdot q=\frac{aq+b}{cq+d}=q^{\prime }\in R\;.$$

Below, we extend SL(2,R) homographic transformations of the line, (15), to SL${}^{\pm }\left(2,R\right)$, the non-connected group of real 2×2 matrices with determinant ±1, in order to include the simple inversion $i_{v}$

(16) $$q\mapsto 1/q=\left(\begin{array}{cc} 0 & 1\\ 1 & 0 \end{array}\right)\cdot x:=i_{v}\cdot x\;.$$

Similarly, we extend SU(1,1) homographic transformations of the circle, (14), to SU${}^{\pm }\left(1,1\right)$ the group of matrices with determinant ±1. We note that the image of (16) under the Cayley transform (13) is the same matrix

(17) $$i_{v}=\left(\begin{array}{cc} 0 & 1\\ 1 & 0 \end{array}\right)\;.$$

## 3. A Subset of Möbius Transformations

We now examine the subset A of SL${}^{\pm }\left(2,R\right)$ made of elements obeying the two constraints:

(i) They are nilpotent,
  (18) $$s^{2}=I\;.$$
(ii) They leave x=1 invariant under Möbius transformations,
  (19) s·1=1.

**Proposition** **1.** *The subset* A *of SL* ${}^{\pm }\left(2,R\right)$ *made of elements obeying (i) and (ii) contains the identity I (up to a sign) and the following one parameter family of* 2×2 *real matrices*

(20) $$\ell \left(a\right)=\left(\begin{array}{cc} a & 1-a\\ 1+a & -a \end{array}\right)\;,\;a\in R\;.$$

**Proof.**  Let us start with a generic element $s=\left(\begin{array}{cc} a & b\\ c & d \end{array}\right)$ in the algebra M${}_{2}\left(R\right)$ with arbitrary determinant Δ=ad−bc. Nilpotence and the condition that 1 is a fixed point under $s\cdot q=\left(aq+b\right){\left(cq+d\right)}^{-1}$ entail the following equations on the matrix elements

(21) $$\begin{array}{cc} a^{2}+bc & =1\;, \end{array}$$

(22) $$\begin{array}{cc} d^{2}+bc & =1\;, \end{array}$$

(23) $$\begin{array}{cc} (a+d)b & =0=(a+d)c\;, \end{array}$$

(24) $$\begin{array}{cc} a+b & =c+d\;. \end{array}$$

The condition Δ=±1 results from the above, of course. (21) and (22) imply $a^{2}=d^{2}$, and so we have the equivalent conditions

(25) $$\begin{array}{cc} a^{2}+bc & =1\;, \end{array}$$

(26) $$\begin{array}{cc} a & =\pm d\;, \end{array}$$

(27) $$\begin{array}{cc} (a+d)b & =0=(a+d)c\;, \end{array}$$

(28) $$\begin{array}{cc} a+b & =c+d\;. \end{array}$$

Case a=d=0
  Then, b=c (from (28)), and $bc=b^{2}=1$ (from (25)). Thus, $s=\pm i_{v}$.
Case a=d≠0
  Then, b=0=c (from (27)), and a=±1=d from (25)). Thus, s=±I.
Case a=−d≠0

Then $bc=1-a^{2}$ (from (25)) and c−b=2a (from (28)), which means that *c* and −b are roots of $X^{2}-2aX+a^{2}-1=0$; i.e, b=1−a and c=1+a, or b=−1−a and c=a−1. These two possibilities yield

$$s=\left(\begin{array}{cc} a & 1-a\\ 1+a & -a \end{array}\right)=\ell \left(a\right)\;,\;\mathrm{or}\;s=\left(\begin{array}{cc} a & -1-a\\ -1+a & -a \end{array}\right)=-\ell \left(-a\right)\;.$$

Since the second one is equivalent, as a Möbius transformation of R, to ℓ(−a), and that a∈R, all possible solutions are ℓ(a) with a∈R, together with the identity *I*, and up to a factor ≠0. □

Consequently, from now on we focus on the particular cases of the map (15); namely,

(29) $$R∋q\mapsto l\left(a\right)\cdot q=\frac{aq+1-a}{(1+a)q-a}\;.$$

Elements of A have interesting properties.

$P_{1}$: Determinant
  (30) detℓ(a)=−1∀a∈R.
  Thus all elements of A, except the identity *I*, have determinant equal to −1.
$P_{2}$: Inverse (from nilpotence)
  (31) $$\ell^{-1}\left(a\right)=\ell \left(a\right)\;\forall a\in R\;.$$
$P_{3}$: Parameter inversion
  (32) $$\ell \left(-a\right)=i_{v}\;\ell \left(a\right)\;i_{v}\;,\;\Leftrightarrow \;i_{v}\;\ell \left(-a\right)=\ell \left(a\right)\;i_{v}\;.$$
$P_{4}$: Particular cases: inversion, affine transformation of the line, and their combination,
  (33) $$\ell \left(0\right)=i_{v}\;,\;\ell \left(-1\right)=\left(\begin{array}{cc} -1 & 2\\ 0 & 1 \end{array}\right)\;,\;\ell \left(1\right)=\left(\begin{array}{cc} 1 & 0\\ 2 & -1 \end{array}\right)=i_{v}\;\ell \left(-1\right)\;i_{v}\;.$$
$P_{5}$: Composition is internal up to inversion
  (34) $$\ell \left(a\right)\;\ell \left(a^{\prime }\right)=\left(\begin{array}{cc} 1+a^{\prime }-a & a-a^{\prime }\\ a^{\prime }-a & 1+a-a^{\prime } \end{array}\right)=i_{v}\ell \left(a^{\prime }-a\right)\;.$$

Hence, since the product of two arbitrary, distinct elements and the difference of the identity has a determinant equal to 1, A is not a subgroup of SL${}^{\pm }\left(2,R\right)$. On the other hand, the subset

(35) $$i_{v}A=\left\{i_{v}\ell \left(a\right)\;,\;a\in R\right\}$$

is an abelian subgroup isomorphic to R. Indeed, it contains *I*, and (31) and (34) imply

(36) $$i_{v}\ell \left(a\right)i_{v}\ell \left(a^{\prime }\right)=i_{v}\ell \left(a+a^{\prime }\right)\;\Leftrightarrow \ell \left(a\right)\ell \left(a^{\prime }\right)=\ell \left(a-a^{\prime }\right)i_{v}\;,$$

which can be directly verified with

(37) $$i_{v}\ell \left(a\right)=\left(\begin{array}{cc} 1+a & -a\\ a & 1-a \end{array}\right)=I+a\varpi \;,\;\varpi =\left(\begin{array}{cc} 1 & -1\\ 1 & -1 \end{array}\right)\;,\;\varpi^{2}=0\;.$$

Let us now write down the counterpart B of A as a subset of SU${}^{\pm }\left(1,1\right)$ by using (13):

(38) $$B=\left\{j\left(a\right)=\left(\begin{array}{cc} -ia & 1+ia\\ 1-ia & ia \end{array}\right)\;,\;a\in R\right\}\;.$$

As a nilpotent homographic transformation of the unit circle, it leaves invariant the point 1.

## 4. Cycles

We now examine specific sequences of maps (29) in view of their relevance to relations between parameters *q* associated with different facets of a complex system.

### 4.1. Two-Term Cycles

Let us consider the two-term cycle

(39) $$q_{1}\mapsto q_{2}=\ell \left(a_{12}\right)\cdot q_{1}\mapsto q_{1}=\ell \left(a_{21}\right)\cdot q_{2}\;,$$

which reads after using (34) and (32),

(40) $$q_{1}=i_{v}\ell \left(a_{12}-a_{21}\right)\cdot q_{1}\;.$$

This leads one to consider the algebraic relation between $a=a_{12}-a_{21}$ and its fixed point $q=i_{v}\ell \left(a\right)\cdot q$; i.e., $\left(1+a\right)q^{2}-\left(1+2a\right)q+a=0$. This equation has two solutions, q=1 (expected), and if a≠−1, q=a/(a+1); i.e., a=q/(1−q). Thus, for arbitrary $q_{1}\neq 1$, (40) does provide non trivial solutions for the doublet $a_{12}$, and $a_{21}$, depending on the initial $q_{1}$,

(41) $$a_{12}-a_{21}=\frac{q_{1}}{1-q_{1}}=\frac{1}{1-q_{1}}-1\;.$$

Let us introduce for our next purposes, the quantities

(42) $$q_{\mathrm{aux}}:=q_{1}\;\mathrm{and}\;a_{\mathrm{aux}}:=\frac{1}{q_{\mathrm{aux}}-1},$$

which are determined by the fixed point. We then get from the above, the “conservation law”:

(43) $$a_{12}-a_{21}+a_{\mathrm{aux}}=-1\;.$$

### 4.2. Three-Term Cycles

Let us now consider the three-term cycle

(44) $$q_{1}\mapsto q_{2}=\ell \left(a_{12}\right)\cdot q_{1}\mapsto q_{3}=\ell \left(a_{23}\right)\cdot q_{2}\mapsto q_{1}=\ell \left(a_{31}\right)\cdot q_{3}\;,$$

which reads after using (34) and (31),

(45) $$q_{1}=\ell \left(a_{31}-a_{23}+a_{12}\right)\cdot q_{1}\;.$$

This leads one to consider the algebraic relation between $a=a_{31}-a_{23}+a_{12}$ and its fixed point q=ℓ(a)·q; i.e., $\left(1+a\right)q^{2}-2aq+a-1=0$. This equation has two solutions, q=1 (expected), and if a≠−1, q=(a−1)/(a+1); i.e., a=(1+q)/(1−q). Thus, for arbitrary $q_{1}\neq 1$, (45) does provide non trivial solutions for the triplet $a_{31}$, $a_{23}$, and $a_{12}$, depending on the initial $q_{1}$,

(46) $$a_{31}-a_{23}+a_{12}=\frac{1+q_{1}}{1-q_{1}}=\frac{1}{1-(1+q_{1})/2}-1\;.$$

Like above, we introduce the quantities

(47) $$q_{\mathrm{aux}}:=\frac{1+q_{1}}{2}\;\mathrm{and}\;a_{\mathrm{aux}}:=\frac{1}{1-q_{\mathrm{aux}}}$$

which are determined by the fixed point. Note the opposite sign of the latter with regard to the previous case. There results the three-term conservation law:

(48) $$a_{31}-a_{23}+a_{12}-a_{\mathrm{aux}}=-1\;.$$

### 4.3. N-Term Cycles

The two above cases allow us to easily infer the general *N*-term case. If N=2p is even, Equation (40) generalises to

(49) $$q_{1}=i_{v}\ell \left(\sum_{i=1}^{N-1}{\left(-1\right)}^{i+1}a_{ii+1}-a_{N1}\right)\cdot q_{1}\;,$$

and yields the fixed point q=a/(a+1), i.e., a=q/(1−q), with $a=\sum_{i=1}^{N-1}{\left(-1\right)}^{i+1}a_{ii+1}-a_{N1}$, and the resulting conservation law

(50) $$\sum_{i=1}^{N-1}{\left(-1\right)}^{i+1}a_{ii+1}-a_{N1}+a_{\mathrm{aux}}=-1\;,$$

where $a_{\mathrm{aux}}:=\frac{1}{q_{\mathrm{aux}}-1}$, $q_{\mathrm{aux}}:=q_{1}$.

If N=2p+1 is odd, Equation (45) generalizes to

(51) $$q_{1}=i_{v}\ell \left(\sum_{i=1}^{N-1}{\left(-1\right)}^{i+1}a_{ii+1}+a_{N1}\right)\cdot q_{1}\;,$$

and yields the fixed point q=(a−1)/(a+1), i.e., a=(1+q)/(1−q), with $a=\sum_{i=1}^{N-1}{\left(-1\right)}^{i+1}a_{ii+1}+a_{N1}$, and the resulting conservation law

(52) $$\sum_{i=1}^{N-1}{\left(-1\right)}^{i+1}a_{ii+1}+a_{N1}-a_{\mathrm{aux}}=-1\;,$$

where $a_{\mathrm{aux}}:=\frac{1}{1-q_{\mathrm{aux}}}$, $q_{\mathrm{aux}}:=\left(1+q_{1}\right)/2$.

Let us finally remark that the extension of these formulas and relations to the case of complex variables is straightforward. The underlying group is SL${}^{\pm }\left(2,C\right)$, the group of conformal transformations of the complex plane or of the (Riemann) sphere.

## 5. With Another Variable

In the previous section, besides the nilpotence, we imposed that a finite point, namely 1, be left invariant. It is instructive to impose now that the point at infinity be left invariant. A conformal transformation which sends 1 to *∞* is given by

(53) $$q\mapsto \tilde{q}=\frac{\lambda }{1-q}=\left(\begin{array}{cc} 0 & \lambda \\ -1 & 1 \end{array}\right)\cdot q\Leftrightarrow q=\frac{1}{\lambda }\left(\begin{array}{cc} 1 & -\lambda \\ 1 & 0 \end{array}\right)\cdot \tilde{q}\;,$$

where λ>0 is a parameter. The Möbius transformation ℓ(a), (20), becomes:

(54) $$\begin{array}{c} {\tilde{q}}^{\prime }=\left(\begin{array}{cc} 0 & \lambda \\ -1 & 1 \end{array}\right)\cdot q^{\prime }=\frac{1}{\lambda }\left(\begin{array}{cc} 0 & \lambda \\ -1 & 1 \end{array}\right)\left(\begin{array}{cc} a & 1-a\\ 1+a & -a \end{array}\right)\left(\begin{array}{cc} 1 & -\lambda \\ 1 & 0 \end{array}\right)\cdot \tilde{q}\\ \;=\left(\begin{array}{cc} 1 & -\lambda (a+1)\\ 0 & -1 \end{array}\right)\cdot \tilde{q}=\left(\begin{array}{cc} -1 & \lambda (a+1)\\ 0 & 1 \end{array}\right)\cdot \tilde{q}\equiv m_{\lambda }\left(a+1\right)\cdot \tilde{q}\;. \end{array}$$

Thus, the corresponding Möbius transformation reduces to a translation combined with the space inversion $\tilde{q}\mapsto -\tilde{q}=i_{s}\cdot \tilde{q}$. By introducing the abelian group $T_{\lambda }$ of translations of the real line,

(55) $$T_{\lambda }=\left\{t_{\lambda }\left(b\right)=\left(\begin{array}{cc} 1 & \lambda b\\ 0 & 1 \end{array}\right)\right\}\;,\;t_{\lambda }\left(b\right)t_{\lambda }\left(b^{\prime }\right)=t_{\lambda }\left(b+b^{\prime }\right)\;,$$

the new transformations read

(56) $$m_{\lambda }\left(b\right)=t_{\lambda }\left(b\right)\;i_{s}=i_{s}\;t_{\lambda }\left(-b\right)\;,\;m_{\lambda }\left(b\right)\;m_{\lambda }\left(b^{\prime }\right)=m_{\lambda }\left(b-b^{\prime }\right)\;i_{s}\;$$

and have similar properties to the ℓ(a)’s above.

Despite the simpler nature of the above geometric operations, we chose in this paper to work with the previous formalism established from the fixed point q=1. Indeed, this value corresponds, in the present context, to the BG particular instance.

## 6. Observational Examples

In this section, we list a series of three-term and two-term cycles issued from various observations.

The first experimental evidence of the existence of a *q*-triplet in nature, conjectured in [17], was achieved in the magnetic fluctuations of the solar plasma, as measured at the Voyager 1 near the end of our planetary system [18]. Our present study is based on this observation. Three deformation parameters, $q_{\mathrm{sensitivity}}\equiv q_{\mathrm{sens}}$, $q_{\mathrm{stationarity}}\equiv q_{\mathrm{stat}}$ and $q_{\mathrm{relaxation}}\equiv q_{\mathrm{rel}}$ are supposed to be part of a three-term cycle of the type described in (44) through the relations

(57) $$\frac{1}{q_{\mathrm{sens}}-1}=:-a_{\mathrm{sens}}\;\;\frac{1}{q_{\mathrm{stat}}-1}=:a_{\mathrm{stat}}\;\;\frac{1}{q_{\mathrm{rel}}-1}=:a_{\mathrm{rel}}\;.$$

This is precisely the guideline in the identification of the parameters $a_{23},a_{31}$ and $a_{12}$ in (44) with these $a_{sen},a_{stat}$ and $a_{rel}$ respectively. Our objective is to reveal a kind of regularity in the sequence of fixed points, or equivalently, of $q_{\mathrm{aux}}$’s, as defined in (47). Furthermore, the two-term cycles are considered if one of the three deformation parameters is equal to 1, which corresponds to the BG statistics. All numerical data are summarised in Table 1.

### 6.1. Observations with Three-Term Cycles

#### 6.1.1. Solar Wind

The following conjectural values are from [19]. By using in situ measurements in the distant heliosphere these authors calculated the *q*-triplet from the magnetic field strength observations made by Voyager 1 at the distance of E40 AU during 1989 and at E85 AU during 2002. Based on these observations, the authors in [19] suggested the emergence of the additive duality 2−q and the multiplicative duality 1/q. To be more precise, it was used the fact that $q_{stat}$ had the smallest error bar so that it was hinted the simple ratio 7/4. Then for the other two *q*-indices, simple rational numbers were once again adopted: see the top of Table 1 (solar wind). In the rest of the table, we follow along a similar path of analysing the *q*-triplets obtained from the various empirical data.

(58) $$\begin{array}{ccc} q_{\mathrm{sens}} & = & -1/2 \end{array}$$

(59) $$\begin{array}{ccc} q_{\mathrm{stat}} & = & 7/4 \end{array}$$

(60) $$\begin{array}{ccc} q_{\mathrm{rel}} & = & 4; \end{array}$$

hence,

(61) $$\begin{array}{ccc} a_{\mathrm{sens}}:=\frac{1}{1-q_{\mathrm{sens}}} & = & 2/3 \end{array}$$

(62) $$\begin{array}{ccc} a_{\mathrm{stat}}:=\frac{1}{q_{\mathrm{stat}}-1} & = & 4/3 \end{array}$$

(63) $$\begin{array}{ccc} a_{\mathrm{rel}}:=\frac{1}{q_{\mathrm{rel}}-1} & = & 1/3; \end{array}$$

hence,

(64) $$\begin{array}{ccc} \frac{1}{q_{\mathrm{rel}}-1} & = & \frac{1}{q_{\mathrm{sens}}-1}+1 \end{array}$$

(65) $$\begin{array}{ccc} \frac{1}{q_{\mathrm{stat}}-1} & = & \frac{1}{q_{\mathrm{sens}}-1}+2 \end{array}$$

One also checks, in order to follow the three-cycle relation (46),

(66) $$a_{\mathrm{rel}}+a_{\mathrm{stat}}-a_{\mathrm{sens}}=1\;,$$

which implies that the fixed point in this case is $q_{1}=0$, and $q_{\mathrm{aux}}=1/2$.

Let us incidentally mention a remarkable relation [32]. If we define ϵ≡1−q, Equations (58–60) are equivalent to

(67) $$\begin{array}{ccc} \epsilon_{\mathrm{sens}} & = & 3/2 \end{array}$$

(68) $$\begin{array}{ccc} \epsilon_{\mathrm{stat}} & = & -3/4 \end{array}$$

(69) $$\begin{array}{ccc} \epsilon_{\mathrm{rel}} & = & -3\;. \end{array}$$

We then verify:

(70) $$\begin{array}{ccc} \epsilon_{\mathrm{stat}} & = & \frac{\epsilon_{\mathrm{sens}}+\epsilon_{\mathrm{rel}}}{2}\;\;\;\;\;\left(\mathrm{arithmetic}\;\mathrm{mean}\right) \end{array}$$

(71) $$\begin{array}{ccc} \epsilon_{\mathrm{sens}} & = & {\left[\epsilon_{\mathrm{stat}}\;\epsilon_{\mathrm{rel}}\right]}^{1/2}\;\;\;\left(\mathrm{geometric}\;\mathrm{mean}\right) \end{array}$$

(72) $$\begin{array}{ccc} \epsilon_{\mathrm{rel}}^{-1} & = & \frac{\epsilon_{\mathrm{stat}}^{-1}+\epsilon_{\mathrm{sens}}^{-1}}{2}\;\;\;\;\left(\mathrm{harmonic}\;\mathrm{mean}\right)\;. \end{array}$$

The possible interpretation of these intriguing relations in terms of some special symmetry, or some analogous property, has proved elusive.

#### 6.1.2. Feigenbaum Point

From [26,27,33,34];

$q_{\mathrm{sens}}$ in [24,25,26];

$q_{\mathrm{rel}}$ in [33,34];

$q_{\mathrm{stat}}$ in [27].

(73) $$\begin{array}{ccc} q_{\mathrm{sens}} & = & 0.2445 \end{array}$$

(74) $$\begin{array}{ccc} q_{\mathrm{stat}} & = & 1.65 \end{array}$$

(75) $$\begin{array}{ccc} q_{\mathrm{rel}} & = & 2.2498; \end{array}$$

hence,

(76) $$\begin{array}{ccc} \frac{1}{1-q_{\mathrm{sens}}} & = & 1.3236 \end{array}$$

(77) $$\begin{array}{ccc} \frac{1}{q_{\mathrm{stat}}-1} & = & 1.5385 \end{array}$$

(78) $$\begin{array}{ccc} \frac{1}{q_{\mathrm{rel}}-1} & = & 0.8001. \end{array}$$

Also, within the error bars, we verify that [35]

(79) $$\epsilon_{rel}+\epsilon_{sen}≃\epsilon_{sen}\epsilon_{stat}\;;$$

hence,

(80) $$q_{\mathrm{rel}}+q_{\mathrm{sens}}-2≃\left[1-q_{\mathrm{sens}}\right]\left[q_{\mathrm{stat}}-1\right]\;.$$

Indeed, $\left[q_{\mathrm{rel}}+q_{\mathrm{sens}}-2\right]/\left[1-q_{\mathrm{sens}}\right]\left[q_{\mathrm{stat}}-1\right]=1.0066$. Notice, however, that this relation does not belong to the set of those that we are discussing in the present paper.

We check in this case that

(81) $$a_{\mathrm{rel}}+a_{\mathrm{stat}}-a_{\mathrm{sens}}=0.8001+1.5385-1.3236=1.015\;,$$

which implies that the fixed point in this case is $q_{1}\approx 0.0075$ and $q_{\mathrm{aux}}\approx 1/2$.

#### 6.1.3. Brazos River

From [28].

(82) $$\begin{array}{ccc} q_{\mathrm{sens}} & = & 0.244 \end{array}$$

(83) $$\begin{array}{ccc} q_{\mathrm{stat}} & = & 1.65 \end{array}$$

(84) $$\begin{array}{ccc} q_{\mathrm{rel}} & = & 2.25; \end{array}$$

hence,

(85) $$\begin{array}{ccc} a_{\mathrm{sens}}=\frac{1}{1-q_{\mathrm{sens}}} & = & 1.323 \end{array}$$

(86) $$\begin{array}{ccc} a_{\mathrm{stat}}=\frac{1}{q_{\mathrm{stat}}-1} & = & 1.538 \end{array}$$

(87) $$\begin{array}{ccc} a_{\mathrm{rel}}=\frac{1}{q_{\mathrm{rel}}-1} & = & 0.8696. \end{array}$$

In this case we have

(88) $$a_{\mathrm{rel}}+a_{\mathrm{stat}}-a_{\mathrm{sens}}=0.8696+1.538-1.323=1.0846\;,$$

which implies that the fixed point in this case is $q_{1}\approx 0.0406$ and $q_{\mathrm{aux}}\approx 0.5203$.

#### 6.1.4. Bitcoin

From [29].

(89) $$\begin{array}{ccc} q_{\mathrm{sens}} & = & 0.14 \end{array}$$

(90) $$\begin{array}{ccc} q_{\mathrm{stat}} & = & 1.54 \end{array}$$

(91) $$\begin{array}{ccc} q_{\mathrm{rel}} & = & 2.25; \end{array}$$

hence,

(92) $$\begin{array}{ccc} a_{\mathrm{sens}}=\frac{1}{1-q_{\mathrm{sens}}} & = & 1.163 \end{array}$$

(93) $$\begin{array}{ccc} a_{\mathrm{stat}}=\frac{1}{q_{\mathrm{stat}}-1} & = & 1.85 \end{array}$$

(94) $$\begin{array}{ccc} a_{\mathrm{rel}}=\frac{1}{q_{\mathrm{rel}}-1} & = & 0.8696. \end{array}$$

In this case we have

(95) $$a_{\mathrm{rel}}+a_{\mathrm{stat}}-a_{\mathrm{sens}}=0.8696+1.85-1.163=1.5566\;,$$

which implies that the fixed point in this case is $q_{1}\approx 0.2176$ and $q_{\mathrm{aux}}\approx 0.6088$.

#### 6.1.5. Standard Map

From [10,30].

(96) $$\begin{array}{ccc} q_{\mathrm{sens}} & = & 0 \end{array}$$

(97) $$\begin{array}{ccc} q_{\mathrm{stat}} & = & 1.935 \end{array}$$

(98) $$\begin{array}{ccc} q_{\mathrm{rel}} & = & 1.4; \end{array}$$

(99) $$\begin{array}{ccc} a_{\mathrm{sens}}=\frac{1}{1-q_{\mathrm{sens}}} & = & 1 \end{array}$$

(100) $$\begin{array}{ccc} a_{\mathrm{stat}}=\frac{1}{q_{\mathrm{stat}}-1} & = & 1.0695 \end{array}$$

(101) $$\begin{array}{ccc} a_{\mathrm{rel}}=\frac{1}{q_{\mathrm{rel}}-1} & = & 2.5. \end{array}$$

In this case we have

(102) $$a_{\mathrm{rel}}+a_{\mathrm{stat}}-a_{\mathrm{sens}}=2.5+1.0695-1=2.5695\;,$$

which implies that the fixed point in this case is $q_{1}\approx 0.4397$ and $q_{\mathrm{aux}}\approx 0.71985$

#### 6.1.6. Ozone Layer

From [31].

(103) $$\begin{array}{ccc} q_{\mathrm{sens}} & = & -8.1 \end{array}$$

(104) $$\begin{array}{ccc} q_{\mathrm{stat}} & = & 1.32 \end{array}$$

(105) $$\begin{array}{ccc} q_{\mathrm{rel}} & = & 1.89; \end{array}$$

hence,

(106) $$\begin{array}{ccc} a_{\mathrm{sens}}=\frac{1}{1-q_{\mathrm{sens}}} & = & 0.11 \end{array}$$

(107) $$\begin{array}{ccc} a_{\mathrm{stat}}=\frac{1}{q_{\mathrm{stat}}-1} & = & 3.125 \end{array}$$

(108) $$\begin{array}{ccc} a_{\mathrm{rel}}=\frac{1}{q_{\mathrm{rel}}-1} & = & 1.12. \end{array}$$

In this case we have

(109) $$a_{\mathrm{rel}}+a_{\mathrm{stat}}-a_{\mathrm{sens}}=1.12+3.125-0.11=4.135\;,$$

which implies that the fixed point in this case is $q_{1}\approx 0.61$ and $q_{\mathrm{aux}}\approx 0.8$.

### 6.2. Observations with Two-Term Cycles

These cycles are degenerate three-term cycles in which one of the *q*’s is =1. For example, for τ=1 day we found that the three solar activity indices—daily sunspot number (SN) from the Sunspot Index Data Center, magnetic field (MF) strength from the National Solar Observatory/Kitt Peak and total solar irradiance (TSI) means from Virgo/SoHO, may be essentially described by the *q*-triplet sets [36] $\left(q_{\mathrm{stat}},q_{\mathrm{sens}},q_{\mathrm{rel}}\right)=\left(1.31\pm 0.07,-0.71\pm 0.10,1\right),\left(1.21\pm 0.06,-0.44\pm 0.07,1\right)$ and (1.54±0.03,−0.52±0.10,1), respectively.

## 7. Conclusions

We have examined a (non exhaustive) series of observational data giving three types of deformation parameter *q*, namely, $q_{\mathrm{sensitivity}}$, $q_{\mathrm{stationarity}}$ and $q_{\mathrm{relaxation}}$, by supposing they are part of an affine three-term cycle, or of a two-term cycle if one of them is 1. Our aim was to establish a kind of conservation law allowing to group the observed phenomena into equivalence classes. In view of our results, one could conjecture that in the case of effective three-term cycles, there exists a class for which the fixed point is $q_{1}\approx 0$. For those cases, we might conjecture that this class of systems with $q_{1}=0$ has only two independent *q*-indices, the third one being given by Equation (66). Other possible *q*-indices, corresponding to other properties, could in principle be obtained by using the present *q*-transformations. In contrast, for the present observations for which $q_{1}\neq 0$ quite neatly, it is premissable to think that yet unobserved values of *q* remain to be included within *q*-quadruplets, or even within higher-order cycles. Something similar could be applicable for the present two-term examples, for which, once again, $q_{1}$ neatly differs from zero. Further progress along the present lines will naturally be very welcome.

## Figures and Tables

**Table 1:** Numerical data from a non exhaustive series of observations displaying sensitivity, stationarity and relaxation *q*-indices, and their respective auxiliary indices issued from three-term cycle or two-term cycle fixed points $q_{1}$. For the three-term cycles, one can observe an interesting closeness between “solar wind” [18,19], “Feigenbaum point” [24,25,26,27] and “Brazos River” [28] in the sense that, for all of them, $q_{1}≃0$, whilst “Bitcoin” [29], “standard map” [10,30], and “ozone layer” [31], are neatly apart from them. With those, as well as with the present two-term cycles, one cannot conclude.

	$q_{\mathrm{sens}}$	$q_{\mathrm{stat}}$	$q_{\mathrm{rel}}$	$q_{\mathrm{aux}}$	$q_{1}$ (Fixed Point)
Solar wind (conjectural)	−1/2	7/4	4	0.5	0
Solar wind (observations)	−0.6±0.2	1.75±0.06	3.8±0.3	0.5158	0.0316
Feigenbaum point (calculations)	0.2444877...	1.65±0.05	2.2497841	0.50375	0.0075
Brazos river (observations)	0.244	1.65	2.25	0.5203	0.0406
Bitcoin (observations)	0.14	1.54	2.25	0.6088	0.2176
Standard map (calculations)	0	1.935	1.4	0.71985	0.4397
Ozone layer (observations)	−8.1	1.32	1.89	0.805	0.61
Solar activity/SN (observations)	−0.71±0.10	1.31±0.07	1	0.725	0.725
Solar activity/MF (observations)	−0.44±0.07	1.21±0.06	1	0.803	0.803
Solar activity/TSI (observations)	−0.52±0.10	1.54±0.03	1	0.544	0.544
